# Sedentary Behavior in People with and without a Chronic Health Condition: How Much, What and When?

**DOI:** 10.3934/publichealth.2016.3.503

**Published:** 2016-08-03

**Authors:** Lucy K. Lewis, Toby Hunt, Marie T. Williams, Coralie English, Tim S. Olds

**Affiliations:** 1 School of Health Sciences, Faculty of Medicine, Nursing and Health Sciences, Flinders University, Adelaide, SA, Australia; 2 Alliance for Research in Exercise, Nutrition and Activity (ARENA), Sansom Institute for Health Research, University of South Australia, Adelaide, SA, Australia; 3 Respiratory Clinical Research Unit, Repatriation General Hospital, Adelaide, SA, Australia; 4 School of Health Sciences, The University of Newcastle, Newcastle, NSW, Australia

**Keywords:** sedentary behavior, chronic obstructive pulmonary disease, stroke, accelerometry, adults

## Abstract

**Purpose:**

To describe sedentary behaviors (duration, bouts and context) in people with and without a chronic health condition.

**Methods:**

Design: Secondary analysis of two cross-sectional studies. Participants: People with stable chronic obstructive pulmonary disease (COPD) (n = 24, male:female 18:6) and their spousal carers (n = 24, 6:18); stroke survivors (n = 24, 16:8) and age- and sex-matched healthy adults (n = 19, 11:8). Level of physiological impairment was measured with post-bronchodilator spirometry (FEV_1_ %predicted) for people with COPD, and walking speed for people with stroke. Outcomes: Participants were monitored over seven days (triaxial accelerometer, Sensewear armband) to obtain objective data on daily sedentary time, and prolonged sedentary bouts (≥ 30 min). During the monitoring period, a 24-hour use of time recall instrument was administered by telephone interview to explore the context of sedentary activities (e.g. television, computer or reading). Sedentary time was quantified using accelerometry and recall data, and group differences were explored. Linear regression examined associations between physiological impairment and sedentary time.

**Results:**

Participant groups were similar in terms of age (COPD 75 ± 8, carers 70 ± 11, stroke 69 ± 10, healthy 73 ± 7 years) and body mass index (COPD 28 ± 4, carers 27 ± 4, stroke 31 ± 4, healthy 26 ± 4 kg.m^−2^). The healthy group had the lowest sedentary time (45% of waking hours), followed by the carer (54%), stroke (60%) and COPD (62%) groups (*p* < 0.0001). Level of physiological impairment was an independent predictor of waking sedentary time (*p* = 0.001).

**Conclusions:**

People with a chronic health condition spent more time sedentary than those without a chronic condition, and there were small but clear differences between groups in the types of activities undertaken during sedentary periods. The study findings may aid in the design of targeted interventions to decrease sedentary time in people with chronic health conditions.

## 1. Introduction

Historically, the term “sedentary” was used to indicate a lack of moderate-to-vigorous physical activity (MVPA). Today, sedentary behavior is defined as the waking time spent in activities that elicit low rates of energy expenditure and characterized by a posture of sitting or lying down [1]. Sedentary behavior has been associated with a number of chronic health conditions including cardiovascular disease, type 2 diabetes, obesity, breast and colon cancer, as well as with cardiovascular and all-cause mortality [2]–[4]. Both the total sedentary time and the pattern in which the time is accumulated appear to be important. Accruing sitting time in shorter bouts may be less detrimental for cardio-metabolic health than prolonged sitting (≥ 30 minutes), even when accounting for levels of MVPA [5],[6].

Sedentary behavior can be measured in a number of ways, including self-reported questionnaires and accelerometer-based activity monitors. The limitations of self-report instruments, such as self-reported time watching television, are well documented [7]. Previous studies have used accelerometry such as the Actigraph GT3X+ device to estimate sedentary time (duration and bouts of sedentary time), with counts of less than 100 per minute defined as sedentary [8]. While accelerometry has been shown to provide a valid measure of sedentary behavior [9], these devices do not provide information regarding the context or types of sedentary behaviors people are engaging in (e.g. using the computer, watching TV or driving in the car).

In older populations where health impairments are evident (e.g. chronic cardiovascular or respiratory disease), there are a growing number of studies exploring associations between use of time, and functional impairment and health outcomes. In people with stroke, sedentary behavior has been evaluated using both posture (time not on feet) [10], accelerometry [11],[12] and energy expenditure [13], while in people with chronic obstructive pulmonary disease (COPD), both tri-axial accelerometers which measure activity counts [14],[15] and Dyna-Port Activity Monitors (DAM) which identify movement patterns and body position have been used [16],[17].

While it is important to identify how much time adults with and without a chronic health condition spend sedentary, it is also valuable to identify the types of activities in which these people engage. Within the broad sedentary behavior domain, there may be some activities that confer positive health outcomes by virtue of their required degree of cognitive (e.g. crossword puzzles) or social engagement (e.g. family get-togethers). Information about the types of sedentary behaviors people engage in may also aid in the development of targeted interventions to reduce sitting time in adults with and without a chronic health condition. Therefore, the aim of this study was to describe the sedentary behavior (duration, bouts and types of activities) in adults with and without a chronic health condition. The chronic health conditions of COPD and stroke were chosen specifically as they are associated with high sedentary time [13],[15], but have differing etiologies and impairments [18].

## 2. Methods

Secondary analyses of two observational cross-sectional studies involving participants with clinically stable COPD and their spousal carers [19], stroke survivors and age- and sex-matched healthy adults [11]. Ethical approvals were granted from the University of South Australia Human Research Ethics Committee (protocol numbers: 0000024007 and 0000023866) and the Southern Adelaide Clinical Human Research Ethics Committee (protocol number: 54/10). Written informed consent was obtained from all participants.

### 2.1. Participants

#### 2.1.1. COPD/Carer Cohort

People with COPD and their spousal carers were recruited from the Repatriation General Hospitals' (RGH) (South Australia) clinical and research databases. People with COPD were included if they had a spirometric diagnosis of COPD according to the Global Initiative for Chronic Obstructive Lung Disease (GOLD) classification [20], had been clinically stable for the four weeks preceding data collection, and lived with a spouse or partner. Carers were defined as a spouse or partner residing with the person with COPD who provided some level of assistance with activities of daily living.

#### 2.1.2. Stroke/Healthy Cohort

People with stroke were recruited from community stroke exercise classes, physiotherapy outpatient services, social media and databases of people discharged from rehabilitation. Healthy control participants were recruited by word of mouth, social media and community exercise classes. People with stroke were included if they were at least six months post-stroke, living at home for at least two months since their stroke, and able to walk independently (with or without walking aids). Healthy control participants were included if they had no prior history of stroke, were the same gender and within five years of age of one of the stroke participants, and did not work more than two days per week in a paid or voluntary capacity.

Cognitive capacity was assessed in each cohort to ensure that participants had sufficient cognitive function to understand study information, and complete the measurement tools. Cognition was assessed using the tool most appropriate for the participant cohort. The Mini Mental State Examination (MMSE) [21] was used with the COPD/carer cohort and the Montreal Cognitive Assessment test (MoCA) [22] was used with the stroke/healthy cohort.

### 2.2. Measurement Procedure

All participants attended a face to face baseline assessment (COPD/carer cohort in a respiratory clinic, stroke/healthy cohort in their own home). Level of physiological impairment was measured with post-bronchodilator spirometry in the COPD/carer cohort, and with walking speed in the stroke/healthy cohort (timing the middle five meters of a nine meter walking course).

### 2.3. Objective Activity Monitoring

Participants were fitted with two activity monitors at the baseline session (Sensewear Pro3^®^ armband, Actigraph GT3X+ accelerometer), and were monitored for seven days, 24 hours a day.

The Sensewear Pro3^®^ armband is a multi-axial, multi-sensor device worn on the dominant upper arm, and has been used previously in people with COPD and stroke [23]–[25]. The sensors in this device measure skin temperature, galvanic skin response, heat flux from the body, and movement [26]. The Sensewear armband has been shown to be a highly accurate measure of sleep when compared to the current gold standard of polysomnography, and is therefore able to distinguish between sleep and reclining [27]. The Sensewear armband only collects data when the sensors are in direct contact with the participant's skin, which allows periods of non-wear of this device to be easily identified. For both cohorts in this study, participants kept a daily sleep/wake and non-wear log. Participants reported identical removal periods for both activity monitors (Sensewear armband and Actigraph accelerometer) in these logs. Therefore, Sensewear armband data were used to evaluate whether the participant was asleep, or non-compliant with activity monitoring.

The Actigraph GT3X+ is a small lightweight triaxial accelerometer which was worn on an elasticized waist band on the right hip. This accelerometer has been used previously in people with COPD and stroke [28]–[30]. Accelerometers in both cohorts recorded activity in 60 second epochs. As per individual study protocols, for the stroke/healthy cohort, participants were required to have at least three valid days (≥ 4 hours wear time) of accelerometry data for inclusion in the analysis. In the COPD/carer cohort, particiants were required to have at least six valid days (≥ 12 hours wear time) for inclusion. Periods determined by Sensewear data as either non-wear or sleep were excluded, leaving only waking wear time minutes for analysis. Sedentary time was classified as <100 counts per minute [31].

### 2.4. Use of Time

Use of time was measured using the Multimedia Activity Recall for Children and Adults (MARCA). The MARCA is a computer-based instrument which uses a structured interview format to record and construct detailed daily activity profiles [19],[32],[33] and has strong test-retest reliability in healthy adults [32] and in people with COPD [19]. Activity profile data is linked to energy estimates [34],[35] and provides a valid estimate of total daily energy expenditature (TDEE) [36]. The MARCA allowed for the identification of discrete activities (e.g. screen time, car travel, reading) within the sedentary behavior domain. The COPD/carer cohort completed two MARCA interviews recalling four days, while the stroke/healthy cohort completed one MARCA interview recalling one randomly chosen day.

### 2.5. Data Analysis

MARCA data were presented as averages of the four recalled days for the COPD/carer cohort and one day for the stroke/healthy cohort. All MARCA primary endpoints were adjusted for age and sex by regressing them against age, fitting a cubic function of best fit and retaining the residuals for analysis. This was done separately according to sex. The residual values for each participant therefore represented deviations from the expected values for age and sex. For clarity, these were back-transformed to actual minutes per day. Radar graphs were drawn to visualise the differences in patterns of time use. Analysis of variance (ANOVA) was used to compare the age- and sex-adjusted values for sedentary behaviors among each of the four groups. Bonferroni sequential corrections were applied to account for multiple comparisons. Fisher's protected least squares difference was used to test for pairwise differences post hoc. With a total sample size of 91, a retrospective assessment of effect sizes showed this design was capable of detecting effect sizes (Cohen's d) of 0.7 with 80 percent power.

Linear regression was completed on each age- and sex-adjusted primary outcome against measures of physiological impairment (FEV_1_ % predicted for the COPD/carer cohort; walking speed for the stroke/healthy cohort) to investigate associations between the sedentary behavior variables (sedentary time and individual activities) and level of physiological impairment. To describe the time of day when participants were sitting, the percentage of participants sitting in each group was calculated at five minute intervals using MARCA data. For clarity, smoothed Lowess curves were plotted (tension = 25).

## 3. Results

A total of 110 participants were recruited (COPD n = 31 and carers n = 31; stroke n = 26 and healthy controls n = 19). There was a 100 percent retention rate for both the Stroke/healthy cohort, and the COPD/carer cohort. Nineteen participants were excluded from the current analysis due to incomplete outcome data (COPD n = 7 and carers n = 7; stroke n = 2 and healthy controls n = 3), resulting in a final sample of 91 participants. The most common missing data were from the activity monitors (n = 13), followed by incomplete MARCA data (n = 2), skin reactions (n = 2), and failure to meet the inclusion criteria for COPD diagnosis (n = 2). There were no differences in age or gender between the included participants (n = 91), and the participants who were excluded from the analysis due to incomplete outcome data (n = 19).

With the exception of the carer group where females comprised the majority of participants, baseline demographics were similar across all groups (Table 1). Both the COPD and stroke groups showed differences in functional impairment (COPD FEV_1_ 54 ± 23% predicted, stroke 0.8 ± 0.4 m.s^−2^) when compared to the non-chronic condition groups (carer 99 ± 24% predicted, healthy 1.4 ± 0.2 m.s^−2^). In addition, a quarter of the stroke group (n = 6) were identified as having some cognitive impairment (MoCA ≤ 21) and spirometry data revealed nearly 20 percent (n = 5) of carers met COPD diagnosis according to the GOLD (2016) criteria despite not identifying themselves as having COPD.

**Table 1.** Mean (SD) values for age, body mass index (BMI), disease status scores and co-morbidities for all participants.

Characteristic	Healthy	Stroke	Carer	COPD
N	19	24	24	24
Age (years)	73 (7)	69 (10)	70 (11)	75 (8)
% Female	42	33	75	25
BMI (kg.m^−2^)	26 (4)	31 (4)	27 (4)	28 (4)
FEV_1_/FVC (%)			76 (9)	48 (13)
FVC (% predicted)			105 (35)	84 (20)
FEV_1_ (% predicted)			99 (24)	54 (23)
Walking speed (m.s^−1^)	1.4 (0.2)	0.8 (0.4)		
MoCA score	26.1 (2.6)	23.2 (4.8)		
MMSE score			28.0 (1.4)	26.8 (5.9)
Co-morbidities (%)*				
Cardiovascular	42	80	63	88
Musculoskeletal	47	24	29	42
Metabolic	11	28	25	17
Respiratory	16	20	8	46
Neurological	5	12	13	4
Vision / hearing	16	16	4	13
Mental health	0	8	13	0
Other	21	12	25	21

FEV_1_ = Forced expiratory volume in 1 second; FVC = Forced vital capacity, MoCA = Montreal Cognitive Assessment, MMSE = Mini-Mental State Examination. *Percentages do not add up to 100 percent as many participants had more than one co-morbidity.

Table 2 shows the age- and sex-adjusted values for the objectively-measured sedentary time variables for the four groups during waking wear time. The mean (SD) waking wear time for the triaxial accelerometers was 930 (50) minutes per day in the healthy group, 995 (76) in the carers, 852 (153) in the stroke group, and 1021 (91) minutes per day in the COPD group. The *p* values presented relate to the main effect across all four groups (ANOVA) with sequential Bonferroni correction, while post hoc analyses identified significant differences between groups. Accelerometer-estimated sedentary time was the lowest in the healthy group (45% of their waking time), followed by the carer (54%), stroke (60%) and COPD (62%) groups.

**Table 2.** Mean (SD) values for objectively-measured sedentary behaviors calculated from Actigraph data (waking wear time), adjusted for age and sex.

Activity	Healthy n = 19	Carer n = 24	Stroke n = 24	COPD n = 24	*p*	Bonferroni-corrected alpha
Sedentary time (min/d)	425 (96) ^1,2^	533 (117) ^1^	517 (128) ^2^	655 (133) ^1^	< 0.0001	< 0.01
Sedentary time (% wake time)	45 (9) ^1,2^	54 (10) ^1^	60 (12) ^2^	62 (12) ^1^	< 0.0001	< 0.01
% in bouts ≥ 30 min	17 (10)	22 (8)	23 (13)	23 (13)	0.22	0.01
SD of bouts (min)	7 (3)	9 (3)	10 (4.1)	10 (4)	0.12	0.01

The *p* value refers to the main effect across all four groups (ANOVA), bold values represent significant differences between groups following sequential Bonferroni correction and values with the same superscript symbols are significantly different from each other; PT: passive transport; SD: standard deviation

Table 3 shows the age- and sex-adjusted values for the self-reported sedentary time variables from the MARCA use of time interview for the four groups. There are consistent differences between the MARCA-estimated and objectively-measured sedentary time in the healthy, stroke and COPD groups, however the MARCA derived data were not-significant following sequential Bonferroni correction. While MARCA estimated sitting time was not identical to accelerometry-measured sedentary time, the two values provided significant overlap.

**Table 3.** Mean (SD) values for self-reported sedentary behaviors calculated from MARCA data, adjusted for age and sex.

Activity	Healthy n=19	Carer n=24	Stroke n=24	COPD n=24		*p*	Bonferroni-corrected alpha
TDEE (MET.min)	2392 (412)^1,2,3^	2097 (209)^1,4^	1894 (300) ^3^	1953 (209)^2,4^		< 0.0001	< 0.01
Sitting time (min/d)*	596 (164)	638 (99)	685 (136)	706 (114)		0.03	< 0.01
Screen (min/d)	295 (145)	278 (94)	314 (181)	322 (146)		0.72	0.03
Television (min/d)	173 (97)	213 (88)	268 (189)	269 (149)		0.07	< 0.01
Computer (min/d)	107 (94)	64 (64)	47 (72)	51 (57)		0.04	< 0.01
Quiet time (min/d)	110 (74)	138 (58)	145 (108)	178 (87)		0.07	0.01
Reading (min/d)	75 (68)	70 (62)	52 (51)	81 (70)		0.43	0.01
Lying awake (min/d)	20 (40)	34 (35)	45 (82)	41 (81)		0.62	0.02
Listening to music (min/d)	11 (24)	21 (25)	28 (66)	3.8 (48)		0.26	0.01
PT (min/d)	37 (28)	40 (23)	41 (52)	45 (21)		0.88	0.05
Eating (min/d)	80 (17)	69 (18)	63 (30)	69 (16)		0.08	0.01
Other (min/d)	87 (98)	122 (75)	135 (82)	106 (62)		0.22	0.01

The *p* value refers to the main effect across all four groups (ANOVA), bold values represent significant differences between groups following sequential Bonferroni correction and values with the same superscript symbols are significantly different from each other; TDEE: total daily energy expenditure; PT: passive transport; SD: standard deviation, * Sitting time (min/d) is slightly lower than the sum of the sedentary super domains (Screen, Quiet time, PT, Eating and Other) due to rounding at the domain level.

**Figure 1.**
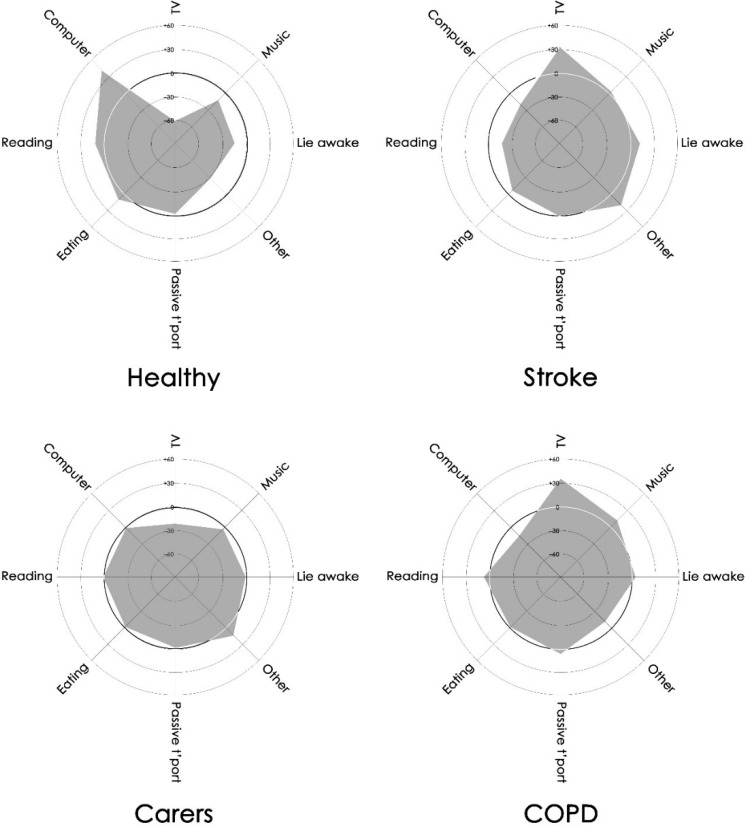
Radar graphs of the sedentary use of time profiles across the four groups. On these graphs, each spoke represents an activity or activity set. The concentric circles represent the time each group spends on each activity relative to the overall mean duration of all participants (dark circle). These range from 60 min/d less than the overall mean (innermost circle) to 60 min/d more than the overall mean (outermost circle). Anything outside the dark circle indicates greater than average time commitment; anything within the dark circle indicates less than average time commitment.

The time use profiles for each group are presented in Figure 1. Differences in time use existed among the groups. The healthy group spent almost one hour more per day using computers than either the stroke or COPD groups. This hour of sedentary activity was more than compensated in the stroke group by more television viewing (95–96 min/d) (*p* = 0.07), and in the COPD group by more quiet time (35–69 min/d) (*p* = 0.07). Only small differences in other types of sedentary behavior were evident. The general shape of the stroke and COPD group profiles were similar.

**Figure 2.**
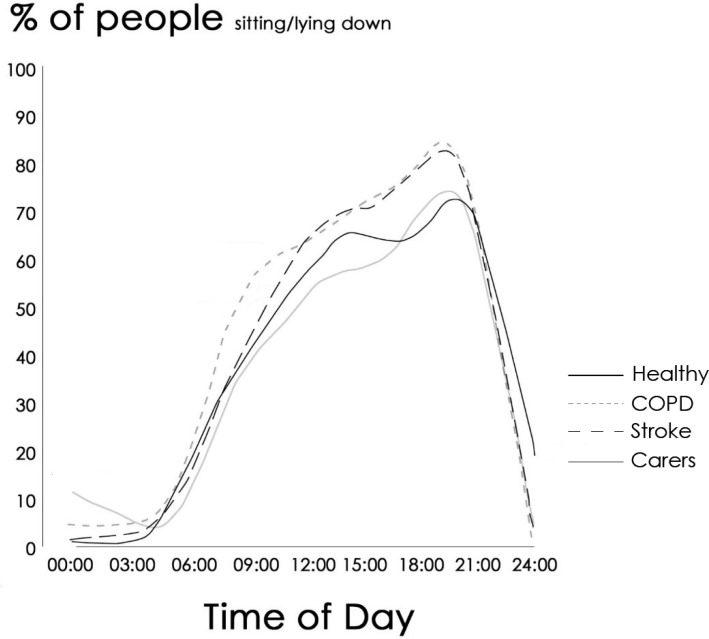
Percentage of participants in each group sitting across the day derived from MARCA interviews. The thick black line represents the Healthy group, the thick grey line the carer group, the dashed line the Stroke group, and the dotted line the COPD group.

Daily distribution of sitting time was assessed using MARCA data. The percentage of each group engaging in seated activities at any given hour of the day, is presented in Figure 2. Similar overall patterns were observed with sharp increases in the number of people sitting after waking, smaller but consistent increases during the middle of the day, and a further increase in sitting during the early evening before people started retiring to bed. Despite these similarities, the stroke and COPD groups consistently had a greater percentage of people sitting at any given hour of the day when compared to the carer or healthy groups.

When regressed against impairment levels (FEV1 % predicted) for COPD and carer groups (Table 4), significant negative associations with the following variables existed: percentage of waking wear time spent sedentary, television watching, and listening to music. There was a significant positive association between FEV1 % predicted and TDEE. Regression against impairment levels in the stroke and healthy groups (walking speed) showed significant negative associations with the following variables: percentage of waking wear time spent sitting, quiet time, lying awake, and listening to music. Significant positive associations were also observed between walking speed and TDEE and computer use.

**Table 4.** Associations between impairment levels (FEV_1_ %predicted for the COPD and carer groups, and walking speed for the stroke and healthy groups) and sedentary variables.

Sedentary variable	COPD/carer cohort		Stroke/healthy cohort	
	FEV_1_ % predicted		Walking speed (m/s)	
	r_s_	*p*	r_s_	*p*
Sedentary time (% wake time)*	−0.46	0.001	−0.49	0.001
% in bouts ≥ 30 min*	−0.11	0.45	−0.14	0.39
SD of bouts (min)*	−0.19	0.21	0.24	0.13
TDEE (MET.min)	0.58	< 0.0001	0.51	0.001
Sitting time (min/d)	−0.17	0.26	−0.27	0.08
Screen (min/d)	−0.28	0.06	0.20	0.20
Television (min/d)	−0.35	0.02	−0.04	0.82
Computer (min/d)	0.15	0.29	0.38	0.01
Quiet time (min/d)	0.05	0.71	−0.35	0.02
Reading (min/d)	0.11	0.45	0.22	0.16
Lying awake (min/d)	0.26	0.07	−0.39	0.01
Listening to music (min/d)	−0.39	0.01	−0.36	0.02
PT (min/d)	−0.08	0.59	−0.27	0.09
Eating (min/d)	0.10	0.51	−0.04	0.78
Other (min/d)	0.11	0.45	−0.27	0.09

* Derived from accelerometry, Bolding denotes a significant association.

## 4. Discussion

The key findings of this analysis of sedentary behaviors observed in similarly aged people with either COPD or stroke, or without chronic conditions were: (1) percentage of waking time spent in sedentary behaviors differed significantly between groups across a continuum (healthy 45%, carer 54%, stroke 60% and COPD 62%); (2) people with COPD or stroke spent significantly more time engaged in sedentary behaviors than otherwise healthy people (carer and healthy groups); (3) people with COPD and stroke spent similar amounts of their waking days in sedentary behaviors; (4) groups did not differ for the percent of waking day spent sitting in bouts of > 30 minutes; and (5) there were small but clear differences between groups in the types of activities undertaken during sedentary periods.

Despite increasing interest in defining activity profiles in people with COPD [16],[28],[30],[37] and stroke [13],[38], previous studies report only general information relating to posture or daily step counts. This study describes sedentary behaviors in terms of duration, bouts of prolonged sedentary time, and types of activities undertaken in four distinct participant groups. We, like others, found that people in the stroke and COPD groups spent significantly more time engaged in sedentary behaviors and there were non-significant trends for these groups to spend more time in prolonged bouts of sedentary behaviors than the healthy control group. These patterns of behavior are of particular concern, given recent findings linking excessive, prolonged sitting time bouts with increased health risks [5],[39]. While the current evidence does not define how much is too much sedentary behavior, nor does it directly prove cause and effect between sedentary behavior and health risks, it does suggest efforts should be made to reduce sedentary behaviors as this may confer important health benefits. Such efforts are particularly important in chronic disease populations where the emergence of comorbid health conditions can compound existing health problems.

Including groups of similar age, with and without a chronic health condition allowed us to explore the association between physiological impairment and sedentary behaviors. While COPD and stroke have differing underlying etiologies and pathophysiology, both chronic health conditions result in a range of impairments but common to both is impaired mobility. Walking speed is used as a sensitive measure of stroke-related disability [26],[40], with links between walking speed and estimates of physical activity level, such as daily step counts. [26]. Similarly, levels of respiratory impairment measured either by maximal voluntary ventilation [41] or Global Initiative for Chronic Obstructive Pulmonary Disease (GOLD) stage [30] are used to quantify severity of airflow limitation and correlate with physical activity estimates such as daily step counts and time spent in MVPA in people with COPD. Unsurprisingly, the presence of physiological impairments independently predicted sedentary behaviors. A surprising finding in the current study was that 20 percent (n = 5) of the carers in the COPD/carer cohort met the COPD diagnosis according to the GOLD (2016) criteria, but did not identify themselves as having COPD. It is possible that shared lifestyle behaviors (including smoking exposure) may have led to the development of a chronic respiratory disease in these spousal carers. It is also possible that their comparatively modest degree of impairment and carer role may have resulted in prioritisation of their partner's medical care / diagnosis, despite experiencing probable signs and symptoms relevant to COPD. This incidental finding may have implications for the screening of family members and social networks for chronic disease.

The drivers for how individuals choose to use their time are complex. For example, inter-relationships exist between activity choice (e.g. housework), posture (standing, walking), and intensity. Exertional dyspnoea and reduced mobility, as observed in people with COPD and stroke respectively, may be one driving force leading these groups to preferentially engage in sedentary behaviors. Leisure time activities may be shared between spousal couples and activity limitations experienced by one person (i.e. people with chronic disease) may impact both spousal members. This may be the reason we found significant differences between carers and healthy controls in the percentage of waking time spent sedentary and TDEE.

Cognitive deficits may have played a role in participants' use of time recall ability as well as activity choice. Around two thirds of stroke survivors have some degree of cognitive impairment [42], while reduced cognitive function is also common in people with COPD [43],[44]. Activities such as reading and computer use are likely to have higher cognitive loads and people with COPD or stroke may preferentially reduce engagement in these activities. Encouraging participation in activities requiring cognitive processing and/or social interaction may confer benefits, given these types of activities have been shown to reduce the risk of developing Alzheimer's disease [45],[46]. The interplay between the detrimental effects of sedentary behaviors and the beneficial effects of cognitively engaging activities is undoubtedly complex and warrants further exploration.

Increasing evidence links specific human behaviors with health outcomes, therefore, understanding not only the type and quantity of activity, but also why certain activities are favored is important and should be considered when developing or evaluating lifestyle modification interventions for people with chronic health conditions. By incorporating use of time interviews such as the MARCA in addition to objective activity monitoring, important insights into older people's activity choices can be identified. Such information may also be useful in better understanding the motivations and/or limitations experienced by different populations.

In this cohort of older adults, 82 percent reported at least one co-morbidity. Conditions of the cardiovascular system were reported most frequently (54% of participants), followed by musculoskeletal (33%), metabolic (22%) and respiratory conditions (20%). Neurological, vision/hearing and mental health complaints were reported by less than 15 percent of participants. While we cannot predict the impact of co-morbid conditions based on the findings of this study, the presence of comorbidities may have influenced our participants' time use choices. Further exploration of the interaction between comorbidities and sedentary behavior is warranted.

### 4.1. Limitations

The findings of the current study are not without limitations. Data included within this secondary analysis were derived from two prospectively planned studies which differed in several study protocol aspects (i.e. duration of accelerometry and MARCA recall). The sample size in each of the four groups was small and sufficient only to detect a large effect size; however, clear trends between the four groups were evident. Several factors may have influenced sedentary time. Firstly, despite demonstrating strong reliability and validity against accelerometry in healthy adults [32], MARCA data may be influenced by poor recall or perceived social expectations. This may particularly be the case for the stroke cohort in which a quarter of the participants had some degree of cognitive impairment. Secondly, we estimated wake time based on Sensewear Pro3® data on the assumption that both activity monitoring devices were worn concurrently. Failure to wear both devices concurrently may have led to inaccuracies in estimations of waking wear time and sedentary behaviors. Additionally, TDEE estimates may have been underestimated as people with chronic conditions have been observed to have increased energy requirements when undertaking everyday activities such as activities of daily living [47],[48] and walking [49]. Disease specific energy expenditure compendia, analogous to Ainsworth's work [34],[35] are lacking, preventing accurate free-living energy expenditure estimates for people with chronic conditions. Finally, day-to-day variability in time use is large and no two days are likely to be constructed identically. The use of a single recall day in the stroke and healthy control cohort may not have accurately represented daily activity profiles in these groups.

## 5. Conclusion

Within aging populations there is an increasing prevalence of chronic health conditions. Emerging research confirms that time use choices can affect health outcomes, independent of physical activity levels. To the best of our knowledge, this is the first exploratory study to describe the time that both healthy people, and people with either COPD or stroke spend in sedentary behaviors, and the type of activities in which they engage. The findings may aid in the design of targeted interventions aimed at modifying individual sedentary activities or to reduce the time spent in generalised sedentary behaviors in these populations.
